# 533 Human Case Characterizations of Skin Burn Using Novel Multi-Spectral Short Wave Infrared Imaging

**DOI:** 10.1093/jbcr/irac012.162

**Published:** 2022-03-23

**Authors:** Johanna H Nunez, Caroline Park, Audra Clark, Chiaka Akarichi, Brett Arnoldo, Samuel P Mandell, Deborah L Carlson, Ryan M Huebinger, Rodney K Chan, Jeremy Goverman, Sergey Mironov, Benjamin Levi, Omer Berenfeld

**Affiliations:** University of Texas, Southwestern, Dallas, Texas; University of Texas Southwestern, Dallas, Texas; University of Texas Southwestern, Dallas, Texas; University of Texas Southwestern, Dallas, Texas; UT Southwestern Medical Center, Dallas, Texas; UT Southwestern, Parkland Regional Burn Center, Dallas, Texas; UT Southwestern Medical Center, Dallas, Texas; UT Southwestern Medical Center, Dallas, Texas; US Army Institute of Surgical Research, San Antonio, Texas; Massachusetts General Hospital, Boston, Massachusetts; University of Michigan, Ann Arbor, Michigan; UT Southwestern, Dallas, Texas; University of Michigan, Ann Arbor, Michigan

## Abstract

**Introduction:**

Determining the depth of skin burns in patients is critical for surgical decision making, but currently lacks accuracy in clinical practice. Short-wave infrared (SWIR) light penetrates tissue more than visual or near-infrared light and is very sensitive to water content. We have shown in animal models that imaging of skin burns in the SWIR range distinguishes between superficial and deep tissue necrosis. Here we present the first 2 cases of multi-spectral SWIR imaging of human burn injury as a first step toward a non-invasive, label-free, technique for burn depth determination.

**Methods:**

Two subjects admitted for mixed depth, thermal, 6% and 7% total body surface area (TBSA), burns were studied. Prior to burn excision, a novel system, based on a specialized camera, imaged the burn areas and normal skin at 4 different SWIR bands. Standard photographs from imaged areas were collected and presented for 5 independent, blinded, surgeons’ assessments. In SWIR images, 3-5 regions of interest (ROIs) were selected in burned and adjacent normal skin and the reflected light intensity in each ROI was averaged.

**Results:**

Visual and SWIR images were collected for 9 burn areas in the hands, arms, and shoulder of 2 patients (Panel A). Fifty ROIs from the burn areas were assessed by the surgeons and 30 (60%) ROIs were agreed as being superficial or superficial partial thickness (n=5), deep partial thickness (n=11), or full thickness (n=14) burns by a majority (60% or above consensus together with a possible disagreement only between deep partial and full thickness burn). In Panel B the cumulative SWIR reflectance intensity at the 4 SWIR bands for the 3 burn groups, determined by expert surgeon evaluation, and normal skin are compared. The reflectance from superficial and superficial partial thickness burns (yellow) were 102.7±1.2%, 102.3±0.7% and 103.4±1.4% of the normal skin reflectance for 1200, 1650 and 1940 nm, respectively. On the other hand, the reflectance from deep partial thickness burns (grey) were 96.7±0.1% and 94.7±0.1%, and for full thickness burn (red) were 96.1±1.4% and 93.7±1.6% of normal skin reflectance for 1650 and 1940 nm, respectively.

**Conclusions:**

We present the first human SWIR study demonstrating a distinct reflectance intensity of SWIR wavelengths for different burn depths based on surgeon assessments. The results motivate further studies of SWIR imaging of burns in the hope to non-invasively and accurately identify operative versus non-operative burns.

